# Primate-ungulate sympatry: a review of ecological interactions and research perspectives

**DOI:** 10.1007/s10329-026-01274-z

**Published:** 2026-07-29

**Authors:** Catarina Coelho, René Bobe, Susana Carvalho

**Affiliations:** 1 Interdisciplinary Centre for Archaeology and the Evolution of Human Behaviour (ICArEHB), Universidade do Algarve, Faro, Portugal; 2 Centro de Investigação em Biodiversidade e Recursos Genéticos, CIBIO/InBIO, Universidade do Porto, Campus de Vairão, Vairão, Porto, Portugal; 3 Department of Science, Gorongosa National Park, Chitengo, Sofala, Mozambique; 4 School of Anthropology and Museum Ethnography, University of Oxford, Oxford, United Kingdom

**Keywords:** Interspecies interactions, Primate-ungulate sympatry, Commensalism, Antagonism, Mutualism.

## Abstract

The co-occurrence of species within the same area raises fundamental questions about how they can persist while sharing space and resources. One of the central aims of community ecology is to understand the processes that allow species to coexist over time. The sympatry between primates and ungulates has not received much specialized attention, despite long-term acknowledgment that the two groups frequently co-occur. In this contribution, we have reviewed and summarized empirical evidence from a set of published studies to determine the nature of primate and ungulate association in space and time, the ecological contexts within which such associations occur, and the types of interactions that have been reported. We particularly examined the trends in the study taxa, the geographic distribution of research, and the functional categories of interactions. Within the literature, interspecific interactions are frequently reported in Africa, between baboons (*Papio* sp.) and small to medium-sized ungulates. Foraging facilitation and antipredator benefits are among the most frequently documented interaction types, typically resulting in commensalistic or asymmetrical mutualistic outcomes favouring ungulate species. Nonetheless, mutualism and antagonistic interactions are also reported, which indicates the context-dependent nature of these relationships. The frequency and form of associations appear to further depend on seasonal variation and habitat structure, although these factors were not consistently taken into account in the studies considered in this review. This synthesis helps establish a set of research priorities to promote more systematic and focused studies of primate-ungulate sympatry.

## Theoretical framework

**Fig. 1:**
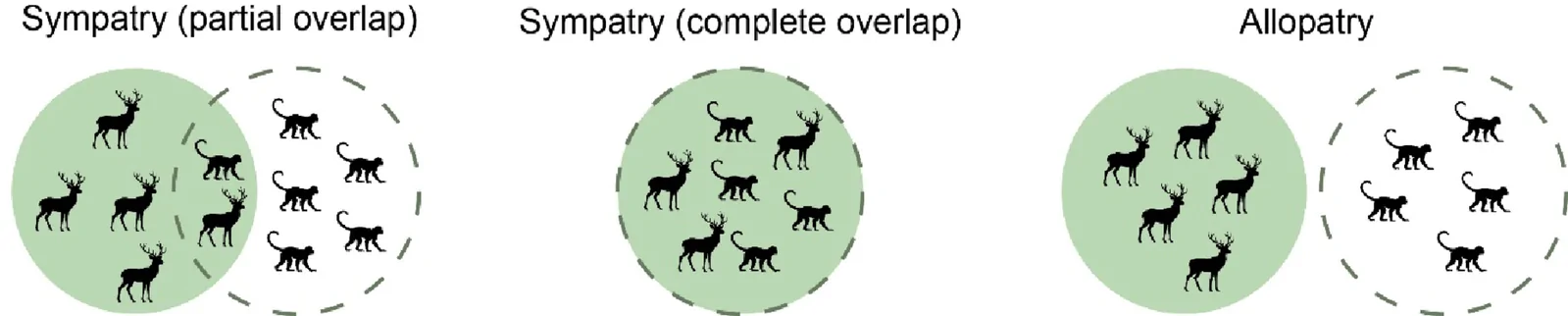
Schematic representation of two species’ distribution. The left and middle panels represent sympatry, with partial and complete range overlap, respectively. On the right, species distributions do not overlap, representing allopatry

Sympatry refers to the presence of two or more species in a common geographic area, where the distribution of either species overlaps, partially or completely. In contrast, when species occur in separate, non-overlapping geographic areas, this is referred to as allopatry (illustrated in Fig. 1) (Mayr 1942; Poulton 1904). Understanding how sympatric species persist over time despite sharing space and potentially competing for similar resources is a central question in community ecology.

This field attempts to describe fluctuations of species richness, abundance, and assemblage, as well as the dynamics by which these fluctuations are created and maintained (Vellend 2010).

One of the most important ecological differences is between habitat, which describes the physical environment an organism occupies, and niche, which refers to the specific way a species lives and interacts with its environment (Chapman and Reiss 1998). The classical theory is based on the idea that every species has its own unique niche, since full overlap would likely result in competitive exclusion, with one species outcompeting the other (Grinnell 1924; Hardin 1960). Nevertheless, overlap in certain dimensions of niche does not always result in exclusion. This concept was formalized in the coexistence theory, which studies how stabilization mechanisms and differences in the competitive ability of species enable them to coexist and maintain species diversity (Chesson 2000, 2018).

Among the mechanisms that support coexistence, there is niche partitioning, which can occur due to dietary preferences, distinct microhabitats, or different activity cycles, reducing direct competition (Becker et al. 2023; Bowers and Brown 1982; Chapman and Reiss 1998; Daskin et al. 2023; Jonathan Davies et al. 2007; Karanth et al. 2017; Kronfeld-Schor and Dayan 2003; MacArthur and Levins 1967; Maire et al. 2012; Pansu et al. 2022; Potter et al. 2022; Schoener 1974). In situations where resources are abundant, there is a possibility of co-occurrence of potential competitor species (Pianka 1974). However, when resources are scarce, competition increases, often leading to other forms of niche differentiation, such as spatial, temporal, or functional segregation, that contribute to the stability of communities (Sladecek et al. 2017; Steinmetz et al. 2021; Vanak et al. 2013; Wiens 1993). Other than competition, other forms of interactions between sympatric species include predation, commensalism, and mutualism, with each having different outcomes for the species involved (Godsoe et al. 2017; Holt 2013).

However, in this context, it is essential to distinguish between interspecific associations and interactions, terms that are often used interchangeably but actually refer to two conceptually distinct phenomena. Interspecific associations are generally defined as repeated spatial or temporal co-occurrence of individuals of different species, which may or may not involve direct behavioural or ecological effects (Stensland et al. 2003; Waser 1982). By contrast, interspecific interactions are processes by which individuals of distinct species influence each other’s behaviour, survival and/or reproduction, affecting individual fitness and community dynamics (Begon et al. 2006; Godsoe et al. 2017). This distinction is particularly relevant because spatial proximity alone does not imply a functional interaction, as it might simply be the result of similar responses to environmental factors. On the other hand, interspecific interactions might be indirect, such that species can influence one another without direct contact or strict spatio-temporal overlap. Moreover, authors commonly attribute functional and potentially evolutionary significance to interspecific associations, often implying benefits for the species involved, such as increased foraging success or increased predator avoidance (Whitesides 1989). However, Whitesides (1989) argued that to attribute functional benefits to interspecies associations, two null hypotheses should first be rejected: (i) observed associations arise by chance and (ii) associations have no effect on the behaviour of the species involved. Together, these concepts provide an important framework for interpreting primate-ungulate sympatry by distinguishing between passive aggregation and biologically meaningful interactions and improving our understanding of how sympatric species coexist over time.

However, the details of the interactions and their outcomes may vary in different taxa and environments, which is why empirical studies should be considered. In the following sections, we review empirical evidence of primate-ungulate sympatry to define the types of interactions described, the taxa involved, and the geographical distributions of study effort, which will serve as a baseline for future dedicated studies of sympatric interactions.

## Empirical cases of primate-ungulate sympatry

### Overview

Mixed-species associations occur across a wide taxonomic range, including between species from different orders, and are most commonly observed among social species (Stensland et al. 2003). Such associations vary in duration, frequency, activity patterns, and structure, depending on the species involved (Cords 1987). Interspecies associations may involve active coordination and direct interactions, passive associations without direct interaction, or aggregations caused by clumped resources rather than attraction between species (Waser 1982). Therefore, as emphasized by Whitesides (1989), such associations should be interpreted cautiously in terms of functional significance.

Primate-ungulate sympatry has been well documented, especially across African savannas and woodlands, where primates and ungulates have coexisted for a long time (Estes 1991; Kingdon et al. 2013; Washburn and DeVore 1961). Despite this long-recognized coexistence, relatively few studies have focused on primate-ungulate sympatry and interspecific interactions, and empirical evidence often result from opportunistic observations. Building on previous reviews that evaluated interspecies associations between primates and other taxa, such as Tsuji (2008), our contribution focuses specifically on primate-ungulate associations as an effort to understand research trends in the field, its limitations and potential directions for future studies.

This paper synthesizes the empirical evidence of primate-ungulate sympatry based on a set of studies, including observational and field-based research conducted across Africa, Asia, and South America over the last six decades. Our review includes studies targeting primate-ungulate associations (e.g. Desbiez et al. 2010; Gurum et al. 1977; Hausfater 1976), as well as broader ecological and behavioural studies in which primate-ungulate interactions were also reported (e.g. Hall 1966; MacKinnon and Mackinnon 1974; Swedell et al. 2008). Altogether, these studies offer a basis for identifying which species are commonly associated, the types of interactions, and broad geographic patterns in the research of primate-ungulate associations. Table 1 provides an overview of the key information from the literature discussed in this review. In the following sections, we examine the patterns of interspecies interactions reported in the literature, as well as potential limitations and implications for future research on the topic. These patterns reflect the studies considered in this review and should, therefore, be interpreted in that context.

### Historical background

As referred above, primate-ungulate sympatry can be set in an evolutionary context with roots in Africa. Owen-Smith (2021) makes the hypothesis that herbivores played a significant ecological role in the development of hominin environments. During Late Miocene and Pliocene, the expansion of more open and seasonal habitats in.

**Table 1:** Summary of reviewed literature documenting interactions between primates and ungulates, including the primate species, associated ungulate species, type of interaction, country of study, and reference

Primate species	Ungulate species	Interaction				Country	Citation
		G	PP	*P*	SI		
*Macaca fuscata yakui*	*Cervus nippon yakushimae*	x				Japan	Agetsuma et al. 2011
*Papio anubis*	*Capra hircus* and *Ovis aries*			x		Nigeria	Akosim et al. 2010
*Semnopithecus sp.*	*Muntiacus muntjak malabaricus*	x				Sri Lanka	Barrette 1977
*Papio anubis*	*Madoqua kirkii* and *Gazella granti*			x		Kenya	Barton 1990
*Papio ursinus*	*Oreotragus oreotragus*			x		Namibia	Davies and Cowlishaw 1996
*Semnopithecus entellus* and *Macaca mulatta*	*Axis axis*	x	x			India	De and Spillett 1966
*Alouatta caraya*	*Pecari tajacu*	x				Brazil	Desbiez et al. 2010
*Papio sp.*	*Tragelaphus scriptus* and *Aepyceros melampus*		x			Kenya	DeVore and Hall 1965
*Chlorocebus pygerythrus* ^1^	*Tragelaphus scriptus*	x				Zambia	Elder and Elder 1970
*Papio ursinus*	*Tragelaphus scriptus*	x				Botswana	Elder and Elder 1970
*Papio anubis* and *Pan troglodytes schweinfurthii*	*Tragelaphus scriptus*			x		Tanzania	Goodall 1986
*Papio anubis*	*Tragelaphus scriptus* and *Aepyceros melampus*				x	Tanzania	Grzimek and Grzimek 1961
*Macaca fuscata*	*Cervus nippon*				x	Japan	Gunst et al. 2018
*Semnopithecus entellus*	*Axis axis*	x	x			India	Gurum et al. 1977
*Erythrocebus patas*	*Ourebia ourebi*		x			Uganda	Hall 1966
*Papio ursinus*	*Aepyceros melampus*			x		Botswana	Hamilton and Busse 1982
*Papio anubis*	*Madoqua kirkii*,* Aepyceros melampus* and *Raphicerus campestris*			x		Kenya	Harding 1973
*Papio cynocephalus*	*Gazella granti*,* Gazella thomsoni* and *Gazella sp.*			x		Kenya	Hausfater 1976
*Chlorocebus tantalus*	*Tragelaphus scriptus*		x			Nigeria	Henshaw 1972
*Papio ursinus*	*Tragelaphus scriptus*	x	x			Rhodesia	Jacobsen 1974
*Semnopithecus entellus*	*Axis axis*		x			India	Jay 1965
*Pongo pygmaeus*	*Sus barbatus*	x				Borneo	Mackinnon 1974
*Semnopithecus entellus*	*Axis axis*	x	x			India	MacKinnon and Mackinnon 1974
*Macaca fuscata yakui*	*Cervus nippon*	x				Japan	Majolo and Ventura 2004
*Semnopithecus dussumieri*	*Tetracerus quadricornis*	x	x			India	Meghwal et al. 2018
*Papio anubis*	*Aepyceros melampus*	x	x			Tanzania	Morgan Davies 1960
*Macaca mulatta*	*Axis axis*	x	x			India	Mukherjea and Gupta 1965
*Semnopithecus entellus*	*Axis axis*	x	x			India	Newton 1989
*Semnopithecus entellus*	*Axis axis*	x	x			India	Rahaman 1973
*Papio cynocephalus*	*Aepyceros melampus*			x		Tanzania	Rhine et al. 1986
*Cebus olivaceus*	*Pecari tajacu*	x				Venezuela	Robinson and Eisenberg 1985
*Semnopithecus entellus*	*Axis axis*	x				India	Schaller 1967
*Papio anubis*	*Tragelaphus scriptus* and *Cephalophus rufilatus*			x		Nigeria	Sommer et al. 2016
*Papio ursinus*	*Oreotragus oreotragus*			x		South Africa	Stoltz and Saayman 1970
*Chlorocebus pygerythrus*	*Equus quagga*,* Phacochoerus aethiopicus*,* Madoqua kirkii*,* Kobus ellipsiprymnus*,* Aepyceros melampus*,* Gazella granti*,* Alcelaphus buselaphus* and *Tragelaphus imberbis*		x			Kenya	Struhsaker 1967
*Papio anubis*	*Gazella thomsoni*,* Madoqua kirkii*,* Raphicerus campestris*,* Aepyceros melampus* and *Oreotragus oreotragus*			x		Kenya	Strum 1975
*Papio hamadryas hamadryas*	*Madoqua kirkii*			x		Ethiopia	Swedell et al. 2008
*Pan troglodytes schweinfurthii*	*Potamochoerus larvatus* and *Tragelaphus scriptus*			x		Tanzania	Teleki 1973
*Papio ursinus*	*Aepyceros melampus*,* Tragelaphus scriptus*,* Kobus ellipsiprymnus*,* Loxodonta africana*,* Phacochoerus aethiopicus*,* Alcelaphus buselaphus lichtensteinii*,* Syncerus caffer caffer*,* Hippotragus niger* and *Cephalophus natalensis*	x				Mozambique	Tinley 1977
*Papio ursinus*	*Connochaetes taurinus*	x	x			Mozambique	Tinley 1977
*Chlorocebus pygerythrus*	*Tragelaphus scriptus*,* Aepyceros melampus*,* Ourebia ourebi* and *Connochaetes taurinus*	unknown				Mozambique	Tinley 1977
*Macaca fuscata*	*Cervus nippon*	x				Japan	Tsuji et al. 2007
*Trachypithecus auratus*	*Rusa timorensis*	x				Indonesia	Tsuji et al. 2015
*Macaca mulatta*	*Rusa unicolor*				x	India	Vasava et al. 2013

Interaction types are coded as follows: *G* : Gleaning, *PP:* Protection against predators, *P:* Predation, and *SI:* Social interaction

Species names were revised to reflect current taxonomy, therefore, nomenclature may differ from the one used in the original publications

^1^The taxonomic update reflects the most probable species for the reported location. However, due to lack of more precise location and proximity to a potential transition zone between two species, we cannot exclude the possibility that the observed individuals correspond to C. cynosuros

Africa was associated with significant faunal turnovers, such as the diversification and abundance of grazing ungulates (Bobe and Behrensmeyer 2004; Owen-Smith 2021). This was also accompanied by the increased terrestriality of primates (Faith and Behrensmeyer 2013; Owen-Smith 2021), which further increased chances of spatial and ecological overlap between primates and ungulates. Koobi Fora records suggest that, through the Plio-Pleistocene, aridification continued and C4 grasslands further expanded, impacting the primary productivity patterns and redistribution of resources, as well as shifting the herbivore guilds to grazing-dominated bovid communities (Bobe and Behrensmeyer 2004; Bobe and Carvalho 2019; Bobe et al. 2022). The herbivores, in turn, affected the vegetation structure, fire regimes, nutrient cycling, and predator distributions, further supporting the description of African savannas as herbivore-driven and predator-rich environments (Owen-Smith 2021).

These ecological pressures likely shaped the evolution of hominins and other primates. In open environments, with less refuge and high carnivore densities, movement and foraging would have been constrained by the risk of predation. Cohesion in groups and collective vigilance may therefore have been favoured under such ecological conditions. Moreover, given the spatial dominance of grazing ungulates documented in Plio-Pleistocene faunal assemblages (Bobe et al. 2022), repeated spatial overlap between primates and ungulates was likely common, potentially generating anti-predator advantages through shared detection of predators, a benefit often suggested for mixed-species groups (Fitzgibbon 1990; Peres 1993; Stensland et al. 2003). Additionally, in these ungulate and predator-rich environments, natural mortality and carnivore kills might have created recurrent opportunities for hominins to access animal matter through scavenging, later evolving into more active forms of hunting, representing early primate-ungulate antagonistic interactions.

Thus, primate-ungulate associations should be viewed not as a recent phenomenon, but as the product of long-standing sympatry in open, predator-rich environments shaped and dominated by herbivores, which likely influenced the ecological, behavioural, and evolutionary trajectories of both groups. At the same time, these associations should also be interpreted considering the ecological conditions that may influence their nature and frequency. In particular, variation in ungulate grazing intensity and its effects on vegetation structure may play a role in primate-ungulate associations. For example, intense ungulate grazing can degrade the vegetation, leading to increased gleaning frequency (Agetsuma et al. 2011). Therefore, primate-ungulate associations should be considered as emerging from long-term evolutionary sympatry, but also within the site-specific ecological context in which they are observed.

### Taxonomic patterns and geographic bias

Baboons (genus *Papio*) appear to be among the most frequently reported primates in association with ungulates. These include olive baboons (*P. anubis*), yellow baboons (*P. cynocephalus*), chacma baboons (*P. ursinus*), and hamadryas baboons (*P. hamadryas*) (e.g. Akosim et al. 2010; Davies and Cowlishaw 1996; Hausfater 1976; Swedell et al. 2008). Moreover, as highlighted by Tinley (1977), baboons play a central role in feeding associations with other species in Gorongosa National Park, Mozambique. This pattern is likely explained by the ecological adaptability of baboons (Beardmore-Herd et al. 2025; Kingdon et al. 2013) and their strong abundance in open and semi-open habitats, such as savanna grasslands and woodlands, where they frequently co-occur with diverse ungulate communities (Falk 2000; Tinley 1977). This is consistent with the fact that many of the empirical studies analysed took place in Africa, where baboons are among the most widespread, ecologically flexible, and abundant primate species (Palombit 2017). In addition to these ecological factors, the prominence of primate-ungulate associations in Africa, particularly involving baboons, may also reflect disproportionate research effort on baboons and other African mammals, which have been more intensively studies compared to other regions and taxa, potentially increasing their representation in the literature. Within the interspecific associations, baboons are frequently reported with bushbuck (*Tragelaphus scriptus*), followed by impala (*Aepyceros melampus*), among other small to medium-sized ungulates (e.g. DeVore and Hall 1965; Jacobsen 1974; Tinley 1977). Bushbucks are typically associated with habitats that provide some cover, such as woodlands (Kingdon et al. 2013). However, they are one of the most widespread antelopes in Africa (East 1999). Impalas are less widespread, occurring predominantly in eastern and southern Africa, but exhibit a high degree of dietary flexibility and adaptability to their environments (Kingdon et al. 2013). As a result, both species are common across African savanna and woodland ecosystems (Mramba 2021; Skinner and Chimimba 2005), increasing the likelihood of overlap and chances for interspecific interactions. The high densities and shared habitat use of these taxa likely contribute to their recurrent associations. Additionally, Tinley (1977) suggests that resource clustering in areas such as the Rift Valley may further contribute to the high frequencies of primate-ungulate associations.

Within the analysed studies, Asia appears to be the second continent with the most reports of primate-ungulate sympatry, particularly for langur-deer and macaque-deer associations (e.g. De and Spillett 1966; Majolo and Ventura 2004; Schaller 1967). Among those, recurrent association occurs in several study sites across India, between the northern plains gray langur (*Semnopithecus entellus*) and the chital deer (*Axis axis*) (e.g., Jay 1965), which is likely a result of their ecological characteristics. *S. entellus* is a highly adaptable, predominantly terrestrial primate distributed across much of the Indian subcontinent, and occupying a wide variety of habitats, including tropical forests, shrublands, woodlands, and anthropogenic landscapes (Kumara et al. 2020; Sayers 2022). Similarly, *A. axis* is a widespread and abundant ungulate in South Asia, commonly found in deciduous forests, adjacent to shrublands and grasslands (Duckworth et al. 2015). The overlapping habitat preferences and high local abundances of both species likely contributes to the high number of reports of this sympatric association. Moreover, the northern plains gray langur is a relatively well studied colobine with several long-term studies (Sayers 2022), which might also contribute to the high number of reports found in the literature here reviewed. Still in Asia, a curious type of interaction was reported in Japan, between *Macaca fuscata* (Japanese macaque) and *Cervus nippon* (sika deer) (e.g., Gunst et al. 2018). Both species are widespread throughout Japan, with increasing populations and expanding distributions. *M. fuscata* is often found in deciduous and evergreen forests (Watanabe and Tokita 2020), and *C. nippon* similarly occurs in forested habitats and woodlands (Harris 2015), indicating broad overlapping habitat preferences that may contribute to their reported association. Besides, the terrestrial habitats of the Japanese macaque may increase its detectability, potentially facilitating the observation of certain types of interactions with the sika deer.

South American cases are comparatively rare in the literature discussed, and document only the association of *Pecari tajacu* (collared peccary) with two species of primates - *Alouatta caraya* (black howler monkey) and *Cebus olivaceus* (wedge-capped capuchin) (Desbiez et al. 2010; Robinson and Eisenberg 1985). *A. caraya* is distributed across most of central-western Brazil, extending into Paraguay, Bolivia, Argentina and Uruguay (Bicca-Marques et al. 2021), while *C. olivaceus* ranges across Venezuela and Guyana, extending to northern Brazil (Boubli et al. 2021). In contrast, *P. tajacu* is broadly distributed across America, from small areas of the United States of America, through Mexico and Central America and much of South America (Gongora et al. 2011). Besides overlapping distributions between the two primate species and the peccary, all three species occur in forested areas (Bicca-Marques et al. 2021; Boubli et al. 2021; Gongora et al. 2011), which contributes to the potential of associations between them. However, both primate species, similarly to most New World monkeys, are predominantly arboreal (Crockett and Eisenberg 1987 Lord 2007; Robinson and Janson 1987), which may reduce their detectability, and help explain the limited number of reported primate-ungulate associations in South America.

These taxonomic and geographic patterns likely reflect the regional differences in the composition and abundance of primate and ungulate communities, species’ ecological traits, and habitat structure, which influence the probability of co-occurrence, as well as the likelihood of detecting these interspecific associations. Additionally, it is important to reinforce that these patterns might also reflect, to some extent, the non-systematic nature of the selection of literature in this review.

### Types of interactions and functional explanations

**Fig. 2:**
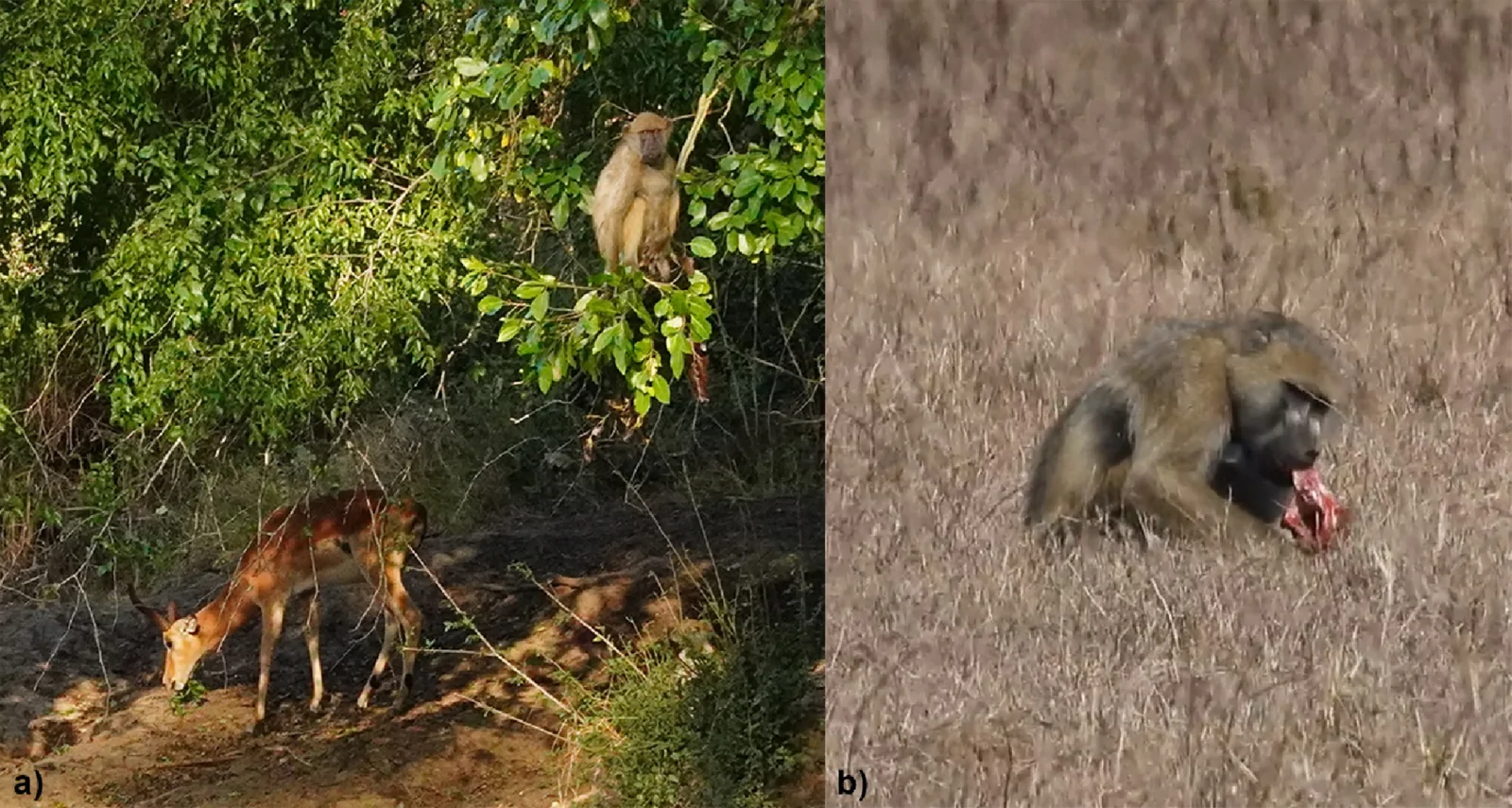
Examples of primate-ungulate interactions from Gorongosa National Park, Mozambique. **a** An impala (A. melampus) gleaning beneath a tree while a baboon (P. ursinus griseipes) feeds above. **b** An adult male baboon consumes a freshly hunted infant reedbuck (Redunca arundinum). [Photographs by Catarina Coelho and Emanuela Rabajoli]

Within the literature, foraging associations appear to be a common mechanism of primate-ungulate sympatry. Through gleaning on food items dropped by primates feeding in trees (illustrated in Fig. 2), ungulates frequently exploit fruits, leaves, flowers, or seeds, gaining access to otherwise unavailable resources (e.g. Elder and Elder 1970; Majolo and Ventura 2004; Robinson and Eisenberg 1985). In most of the studies analysed, these interactions have been interpreted as cases of commensalism or asymmetrical mutualism, benefiting only the ungulate species.

Among these studies, some provide evidence that gleaning rates change across seasons, suggesting that seasonality might play a role in foraging interspecific associations (Agetsuma et al. 2011; Gurum et al. 1977; Robinson and.

Eisenberg 1985; Tsuji et al. 2007, 2015). In particular, higher frequencies of gleaning were reported during periods of food scarcity for ungulates, pointing out that seasonal variation in this behaviour might be linked to food availability (Gurum et al. 1977; Robinson and Eisenberg 1985; Tsuji et al. 2007, 2015). Furthermore, Tsuji et al. (2007) proposes that increased nutritional demands of the sika deer during spring may further explain higher gleaning rates. Altogether, these findings appear to suggest that foraging associations might help mitigate limited resource availability and provide access to more nutritious food items. Another important highlight comes from Newton (1989), who observed that chital deer use cues from langurs, such as movement in the tree canopy and leaves dropping, to initiate the associations. Similarly, Tinley (1977) reported that sounds associated with baboons or falling fruits often lead ungulate species, such as elephants, bushbuck, impala and red duiker, to move under the trees where the baboons are feeding and start consuming the fallen fruits, as well as following them from tree to tree. In addition, Desbiez et al. (2010) observed peccaries moving close to and away from the trees simultaneously with howler monkeys. These reports suggest that foraging associations may be intentional, rather than the result of chance encounters between species.

Another mechanism often described in the literature for primate-ungulate sympatry is anti-predator strategy, which includes increased vigilance, facilitated predator detection, and eavesdropping on alarm calls. Several studies report ungulates exploiting primate alarm calls, with primates often alerting ungulates more frequently than vice versa (e.g. De and Spillett 1966; Elder and Elder 1970; Heymann and Hsia 2015; Meghwal et al. 2018), and ungulates also reported to be less vigilant in the presence of primates (Struhsaker 1967). More specifically, solitary male wildebeests (*Connochaetes taurinus*) have been reported to benefit from baboon vigilance, which may provide potential indirect foraging advantages by reducing the need for vigilance and consequently increasing the time available for feeding (Tinley 1977). However, the opposite pattern has also been noted, namely for the patas monkey (*Erythrocebus patas*) apparently reacting to oribi’s (Ourebia ourebi) alarm calls (Hall 1966) as well as langurs (*S. entellus*) being alerted by chital deer vocalizations (Jay 1965; Rahaman 1973). These types of interaction also fall under the commensalism or asymmetrical mutualism category and might facilitate the use of habitats with higher predation risk by ungulates, when primates are present (Struhsaker 1967; Washburn and DeVore 1961). In particular, this was noted by Morgan-Davies (1960) in Tanzania, where impalas use areas of tall vegetation, usually avoided, when in the presence of baboons. Contrasting with these largely asymmetrical associations, some studies described reciprocal antipredator relationships, in which both primates and ungulates respond to each other’s alarm calls, suggesting a mutualistic outcome (DeVore and Hall 1965; Struhsaker 1967).

Beyond facilitative and mutualistic interactions, primate-ungulate sympatry may also involve direct antagonism, including ungulate predation by primates (Fig. 2). Multiple studies report baboons killing and consuming a variety of ungulate species (e.g., gazelles, dik-dik, impala, bushbuck), typically infants or juveniles (e.g. Barton 1990; Davies and Cowlishaw 1996; Hausfater 1976; Strum 1975). These reports are mainly described as opportunistic rather than intentional and active search for prey (Harding 1973; Hausfater 1976; Sommer et al. 2016). However, some cases report occasional chasing of prey (Hamilton and Busse 1982) as well as more systematic predation, involving hunting and meat sharing (Strum 1975). It is not surprising that baboons are commonly reported in predation events, given their generalist diets (DeVore and Hall 1965; Whiten et al. 1991), however Teleki (1973) and Goodall (1986) also documented these types of events in chimpanzees (*Pan troglodytes schweinfurthii*). Two other studies also indicate an apparent influence of seasonality in the predation events. According to Harding (1973), predation occurs more frequently during the long dry season, which might be related to the scarcity of food resources, making baboons look for additional sources of nutrients. Additionally, predation appears to also be more frequent during the calving periods of the prey species (Sommer et al. 2016), which reveals once more the opportunistic nature of these interactions.

### Interspecific social interactions

A fourth form of primate-ungulate interaction described in the literature is interspecific social behaviours. These apparently rare but remarkable observations merit separate discussion given their implications for interspecific tolerance and social complexity.

Within the studies analysed in our review, interspecies social behaviours have been documented in only a limited number of species pairs, including olive baboons engaging in social play with bushbuck and impala in Serengeti (Grzimek and Grzimek 1961), Japanese macaques mounting and grooming sika deer in Japan (Gunst et al. 2018), and rhesus macaques (*Macaca mulatta*) engaging in grooming and riding behaviours with sambar deer (*Rusa unicolor*) in India (Vasava et al. 2013).

Among these systems, the association between Japanese macaques and sika deer has been the focus of recent research and has been described as a case of co-culture, suggesting that prolonged coexistence and repeated interactions can give rise to culturally transmitted behaviours (Sueur and Huffman 2024). The authors suggested that this interspecies association appears to have originated as a feeding interaction, with deer foraging beneath trees while macaques were feeding. In the early stages, this association was only benefiting the deer but shifted from accidental to more regularized encounters when deer began tracking down the macaques, which is consistent with a learning evolutionary process. At the same time, macaques seem to have evolved to increasingly tolerate the presence of deer and ultimately engage in rare interspecific social behaviours, such as grooming, resting in close contact, play, and mounting (Majolo and Ventura 2004; Sueur and Huffman 2024), which are known to be a core component of intraspecies interactions, playing a key role in dominance, reproduction, and bonding (de Waal 1986). Notably, most of these interactions involved younger individuals, which might suggest that interspecific behaviours may be biased towards life stages characterized by social learning and skill acquisition, especially in highly social species like primates (Whiten and van de Waal 2018). Such interactions may provide opportunities for primates to practice social behaviours and explore social boundaries, potentially contributing to the development of essential skills for their species.

Because such interspecific social behaviours are rare and restricted to a small number of systems, this case should be interpreted as a model system for exploring the limits of interspecific sociality, rather than as a generalizable pattern across sympatric communities. Altogether, this system illustrates how repeated ecological associations may develop into more complex forms of interspecific tolerance and social interaction.

### Limitations and new perspectives

Africa appears to be the most represented region in the empirical reports of primate-ungulate sympatry here reviewed (e.g. Elder and Elder 1970; Harding 1973; Hausfater 1976; Sommer et al. 2016; Strum 1975). This geographic bias likely reflects: (i) the high diversity and extensive sympatry of primates and ungulates in African savannas and woodlands (Kingdon et al. 2013; Tinley 1977), (ii) the presence of long-term primate field sites with extensive observational records (e.g., Amboseli National Park and Gombe National Park), and (iii) the open and conspicuous nature of many savanna and woodland landscapes, which likely facilitates the detection and documentation of interspecific interactions (Bourlière 1985; Newton 1989; Tinley 1977). To add to this, there is also a strong observational bias towards highly terrestrial, diurnal, and group-living primates, particularly baboons. Altogether, this may suggest that these primate-ungulate associations are more common, while in fact they may solely reflect the methodological and detection bias rather than indicating a true absence of such interactions among other species and habitats.

A major limitation of the studies analysed is the lack of standardized definitions for what is considered an interspecific association. Most of these studies leave the definition implicit, differing from simple spatial co-occurrence, repeated proximity, or coordinated movement. Besides that, the mechanisms by which interspecies associations arise vary, as they may result from chance encounters, independent responses to the same stimulus, or from active tracking or following of one species by another (Newton 1989; Waser 1982). However, the distinction between passive aggregation and true associations is often difficult as well (Desbiez et al. 2010), although some of the literature discussed in this paper provides evidence for active tracking and the repeated maintenance of associations rather than passive co-occurrence (Majolo and Ventura 2004; Newton 1989). The inconsistency of definitions makes cross-study comparisons difficult and highlights the need for greater conceptual and methodological standardization in future research.

Although some studies have shown that seasonality may play a role in the frequency and nature of interspecific associations (Gurum et al. 1977; Robinson and Eisenberg 1985; Tsuji et al. 2007, 2015), this was not systematically accounted for, again limiting further conclusions to be taken about its importance in primate-ungulate sympatry. Moreover, many studies fail to provide and control ecological and social covariates, such as primate and ungulate group sizes, habitat structure, predator density, and seasonality. This likely derives from the fact that most studies reported interspecific interactions opportunistically, rather than being specifically designed to investigate primate-ungulate sympatry. As a result, it might limit the ability to assess causal mechanisms and temporal dynamics of interspecies associations, highlighting the need for dedicated, long-term research frameworks.

The empirical evidence discussed highlights that primate-ungulate sympatry is commonly characterized by facilitated foraging, through gleaning, and anti-predator benefits, with ungulates generally deriving greater advantages from such associations (Heymann and Hsia 2015). These interactions are typically classified as commensalistic or asymmetrical mutualistic, reflecting unequal benefits, even though mutualism was also documented in some cases. However, antagonistic interactions, such as predation by primates on ungulates, are also well documented. These observations highlight that primate-ungulate relationships may shift along a continuum from facilitation to antagonism depending on the ecological context. Thus, an important approach for future research is to examine how such antagonistic dynamics are mitigated to allow for the persistence of commensalistic or mutualistic associations within the same systems. In addition, the reports of predation may be disproportionately represented in the literature because several of the reviewed studies were focused on the diet. Moreover, the predominance of predation on infant ungulates suggests that such events are dependent on the size and vulnerability of prey, reinforcing the interpretation of these interactions as largely opportunistic.

Finally, recent work on Japanese macaques and sika deer has suggested that some primate-ungulate systems may involve gradual processes that result in socially complex interactions (Sueur and Huffman 2024). This case points out the importance of long-term, site-based studies for evaluating the potential role of learning, co-adaptation, and co-evolution in shaping primate-ungulate sympatry.

Altogether, the studies considered in this synthesis provide a good record of behavioural direct interactions between species. However, an important component of sympatry was largely overlooked, which refers to niche overlap. Most of these studies focused on visible interspecific associations and interactions, yet sympatry is also shaped by the extent to which species share space, diet and temporal activity, even if they don’t necessarily engage in any direct interactions. Therefore, quantifying niche overlap can provide critical insights into how primates and ungulates partition or share the habitat and resources, and how potential competition is mitigated in these persistent associations. This data may provide important ecological and evolutionary context to primate-ungulate sympatry, especially when long-term datasets are complemented by paleoenvironmental and fossil evidence, allowing these contemporary associations to be interpreted in the light of historical community assemblies, faunal turnover, coexistence and environmental change.

A further advancement in primate-ungulate sympatry studies would be to adopt the framework proposed by Whitesides (1989), in which observed patterns of interspecific associations are tested against null hypotheses. The first being that associations occur as a result of chance encounters, and the second that associations have no effect on the behaviour of the animals involved. To test the first, Whitesides has used both the gas model (Waser 1982) and computer simulations to estimate the probability of chance encounters between independently moving species, based on their densities, movement patterns and spatial use. These predicted encounter rates and durations can then be compared with the observed association rates derived from quantified field observations. However, even if associations do not occur more frequently or for longer than predicted by chance, they can still have biological significance, and the functional implications can be further explored by assessing if behavioural patterns differ when individuals are in association versus when they are not. In addition, Whitesides (1989) highlights the importance of considering temporal changes in interspecific associations, underscoring the need for long-term datasets. Following his approach, new studies could draw more robust inferences regarding the functional advantages and possible evolutionary causes of interspecies associations.

More generally, methodologically speaking, the studies reviewed were based on direct observation. While this method remains fundamental, it may result in an underestimation of the frequency and diversity of primate-ungulate interactions, due to observer effects and detection limitations. As pointed out by Majolo and Ventura (2004), ungulates can easily be alarmed by the presence and proximity of human observers, which might deter them from approaching and engaging in interspecific interactions with primates, instead fleeing away from the observers. Therefore, a big improvement for future studies would be to integrate additional and complementary methodologies. Camera traps, for example, allow continuous and non-invasive monitoring that could be used to complement observational data, by providing additional records of interactions that escape the observer’s eye, including more cryptic associations or rarer types of interactions. In light of Whitesides (1989) framework, such approaches could contribute to more complete behavioural and quantitative datasets, which are essential to infer the ecological advantages of interspecies associations. Similarly, playback trials can be used to assess interspecific responses to alarm calls and predator cues, to further investigate the potential function of primate-ungulate associations.

In addition, GPS radio collaring allows for fine-scale analysis of spatio-temporal overlap between species. This can be particularly helpful when the null hypothesis that observed associations do not exceed rates predicted by chance cannot be rejected, because it does not prove the hypothesis is correct (Whitesides 1989). Therefore, using spatial data allows the analysis of movement patterns of species and can help distinguishing between chance encounters and intentional associations. When combined with accelerometer sensors, GPS collars also enable the classification of behavioural stages (Roberts et al. 2016), which can inform about activity patterns and contribute to a more comprehensive dataset. Finally, to obtain a clearer assessment of niche overlap, dietary analyses, such as DNA metabarcoding, should also be included to quantify the overlap in resource use and better evaluate the extent to which sympatric primates and ungulates share or partition food resources. Moreover, dietary composition and nutritional analyses may help assess potential foraging advantages of interspecific associations, particularly those involving gleaning, by evaluating whether individuals gain access to otherwise unavailable food items or higher-quality food resources.

Together, these methodologies, allied to long-term data collection and hypothesis-driven studies, can strongly improve our understanding of primate-ungulate sympatry. This integrative framework can improve the detection, quantification and interpretation of primate-ungulate associations, contributing to a shift in research on the topic from anecdotal observations to systematic studies, ultimately allowing a deeper understanding of the ecological significance of these associations.
